# The Influence of Composition and Manufacturing Approach on the Physical and Rehydration Properties of Milk Protein Concentrate Powders

**DOI:** 10.3390/foods9020236

**Published:** 2020-02-22

**Authors:** David J. McSweeney, Valentyn Maidannyk, Sharon Montgomery, James A. O’Mahony, Noel A. McCarthy

**Affiliations:** 1 Food Chemistry and Technology Department, Teagasc Food Research Centre, P61 C996 Fermoy, Ireland; David.mcsweeney@teagasc.ie (D.J.M.); Valentyn.Maidannyk@teagasc.ie (V.M.); Sharon.montgomery@teagasc.ie (S.M.); 2 School of Food and Nutritional Sciences, University College Cork, T12 K8AF Cork, Ireland; sa.omahony@ucc.ie

**Keywords:** milk protein concentrate powder, spray drying, rehydration, solubility

## Abstract

This study investigated the physical and rehydration properties of milk protein concentrate (MPC) powders with five different protein contents (i.e., 38.9, 53.7, 63.6, 74.1, and 84.7%, *w*/*w*) prepared by recombining the ultrafiltration (UF) retentate and UF permeate of skim milk. Powder density and flowability increased, while the powder particle size decreased with decreasing powder protein content. The amount of non-wetting MPC powder decreased with decreasing protein content, demonstrating greater wettability for lower protein powders. At protein contents >65% (*w*/*w*), the dispersibility and solubility of the powders decreased significantly, likely due to the greater hydrophobic interactions between casein proteins and a lower concentration of lactose. Therefore, as the protein content of the MPC powders was decreased, their rehydration properties improved. The results obtained in this study provide novel insights into the relationship between the composition of recombined UF retentate and UF permeate streams on the subsequent powder particle size, density, and rehydration properties, and demonstrate that such powders possess similar properties to those prepared using conventional direct membrane filtration.

## 1. Introduction

The global demand for milk protein ingredients has increased greatly in recent years due to increased consumer awareness of the health benefits and importance of dietary protein as well as the economic development of countries in Europe and Asia [1]. Milk protein concentrate (MPC) ingredients are produced through the ultrafiltration (UF) of skim milk, followed by diafiltration to remove additional lactose and other low molecular weight material (i.e., to increase the protein content) before water removal through the use of evaporation and spray drying [2,3,4]. MPC generally contains 40–80% protein [5] and possesses the same ratio of casein to whey as found in skim milk (i.e., ~80:20). The quantity of lactose, minerals, and water in the skim milk decreases as the protein content increases during membrane filtration [6]. The permeate stream generated from this process (i.e., the milk components that pass through the membrane) is collectively referred to as milk permeate.

The applications of MPC include infant milk formula, cheese, yogurt, and products designed for sports and medical nutrition; however, its uses are often limited by its inherent poor solubility [7,8]. This is associated with the presence of insoluble material formed by non-covalent (hydrophobic) protein–protein interactions that occur during the powder manufacturing process and subsequent storage. Therefore, hydration and dissolution of MPC powders is usually conducted in water at approximately 50 °C [9], whereby the increase in solvent temperature accelerates the release of material from the powder particles into the aqueous phase [10]. In order to ensure complete protein hydration, solutions may need to be cooled to 4 °C in order to reduce hydrophobic interactions between casein micelles and allow full hydration and swelling to occur. Furthermore, other high protein dairy powders such as micellar casein concentrate, which is produced by the microfiltration of defatted milk and consists predominantly of casein proteins, also exhibits poor reconstitution properties [11,12]. Such rehydration challenges are compounded when powders are exposed to unfavourable environmental conditions such as high temperature and high relative humidity [13,14,15,16]. The deterioration in solubility over time has been attributed to the presence of cross-linked casein micelles at the surface of the powder particles, which can reduce the transfer of water and thus inhibit dissolution [9,17]. Rehydration of casein-dominant powder is characterised by five stages: (a) wetting, (b) swelling, (c) sinking, (d) dispersion, and (e) dissolution [18]. These steps can be influenced by several factors: (i) pre-treatment of the concentrate (e.g., using high shear) [19], (ii) processing conditions such as spray drying temperatures [20], and (iii) the relative humidity and temperature at which the powder is stored [21]. Furthermore, the powder surface composition (e.g., presence of fat), particle structure (e.g., porosity), and rehydration conditions (e.g., stirring rate and solvent temperature) also play important roles in powder dissolution [22,23].

The standardisation of high protein dairy concentrates through the addition of milk permeate to UF retentate could allow for a precise and efficient approach to manufacture targeted MPC ingredients with a wide range of compositions. Therefore, the aim of this study was to first determine the influence of the protein content of MPC powders, prepared from blends of UF retentate and UF permeate, on the powder density, air content, particle size, flowability, microstructural properties, and subsequent powder rehydration. Second, these results were compared to previous studies from the literature that assessed high protein dairy (mainly MPC) powders produced via conventional direct UF, without the addition of milk permeate, to determine whether or not this novel manufacturing approach would produce powders with the same properties.

## 2. Materials and Methods

### 2.1. Manufacture of Milk Protein Concentrate Powders

Milk protein concentrate (MPC) powders were produced in the Bio-functional Food Engineering Facility at Teagasc Food Research Centre (Moorepark, Fermoy, Co. Cork, Ireland) using a similar method as that described by Maidannyk [24]. Liquid MPC (19.5 and 16.6% *w*/*w*, total solids, and protein, respectively; i.e., MPC85) and concentrated milk permeate (24% *w*/*w*, total solids) were obtained from a local dairy supplier directly after ultrafiltration (UF) and reverse osmosis, respectively. Milk permeate was then combined with the UF retentate to dilute the protein content to ~75, 65, 55, and 40% *w*/*w*, protein. The subsequent five (i.e., MPC85, 75, 65, 55, and 40) MPC batches were stored overnight at 4 °C under gentle agitation. MPC batches were then pre-heated to 45 °C and spray dried using a single-stage spray dryer (Anhydro F1 Lab Dryer; Copenhagen, Denmark) equipped with a two-fluid nozzle atomisation system (Type 1/8 JAC 316ss) under counter-flow drying conditions. The atomisation pressure was set at ~2–3 bar. Air inlet and outlet temperatures were maintained at 185 and 85 °C, respectively. After spray drying, powders were stored in polyethylene plastic bags at 4 °C for the duration of the study.

### 2.2. Compositional Analysis of Milk Protein Concentrate Powders

The free moisture and ash content of the MPC powders was determined using a TGA701 thermogravimetric analyser (LECO Corporation, St Joseph, MI, USA). The protein nitrogen values of the MPC powders were obtained by the Dumas method using a LECO FP628 nitrogen analyser (LECO Corporation, St Joseph, MI, USA); the protein content was determined by multiplying the nitrogen concentration by a nitrogen-to-milk protein conversion factor of 6.38. The fat content of the MPC powders was analysed using the Rose Gottlieb method [25]. The lactose contents were calculated by difference. All analysis was carried out in triplicate, except for fat determination, which was performed in duplicate.

### 2.3. Bulk Density, Particle Density, Occluded, and Interstitial Air

The loose and tapped (100 taps) bulk density of the MPC powders were measured as per GEA Niro [26] using a jolting volumeter STAV II (Funke Gerber, Berlin, Germany). Particle density of MPC powders was measured using an AccuPyc II 1340 gas pycnometer (Micromeritics Instrument Corporation, Norcross, GA, USA), according to the air pycnometer method of GEA Niro [27]. The volume of interstitial air and occluded air was calculated as outlined in the GEA Niro method [27].

### 2.4. Powder Particle Size Distribution

The particle size of the MPC powders was determined using a Malvern Mastersizer (Mastersizer 3000; Malvern Instruments Ltd, Malvern, Worcestershire, UK) equipped with an Aero S dry dispersion unit. The refractive index was set at 1.45. The air pressure was set at 2 bar for all samples, and the feed rate was adjusted (from 25–100%), depending on the cohesiveness of the sample. Size measurements were recorded as the median diameter (D_50_) and cumulative diameters (D_90_ and D_10_) whereby 50, 90, and 10% of the powder volume is represented by powder particles smaller than the size indicated. The volume weighted mean particle diameter (D_[4,3]_) was also calculated.

### 2.5. Powder Flowability and Compressibility

A Powder Flow Tester (PFT; Brookfield Engineering Laboratories, Inc., Middleboro, MA, USA) was used to measure the flowability, bulk density, and compressibility of the MPC powders. Samples were prepared for analysis by filling each into an aluminium trough (volume of 230 cm^3^, 15.2 cm internal diameter). A curved blade was then used to bring the powder into the required conformation for flow function testing and a vane lid was attached to the compression plate before testing. Samples were analysed in triplicate.

A flow function (FF) test was carried out to determine the flowability of the MPC powders. This involved applying five normal stresses (1.0, 1.9, 2.9, 3.9, and 4.8 kPa) and three over-consolidation stresses at each normal stress. A FF graph was obtained by plotting major principal consolidating stress (MPCS) as a function of unconfined failure strength (UFS). This corresponds to the strength that develops within a powder when consolidated, which must be overcome to enable powder flow [28]. Flow index (i) values were calculated from the inverse of the slope of the FF curve. Loose bulk density (p*_b_*) and tapped bulk density (p*_t_*) were recorded at minimum and maximum MPCS, respectively. The Hausner ratio was calculated by dividing the tapped or compressed bulk density by the loose bulk density. The compressibility index (Equation (1)) was calculated as the percentage increase from the loose bulk density to tapped bulk density [29]:

(1) $$C=\frac{p_{t\;}-\;p_{b}}{p_{t}}\;\times \;100$$

### 2.6. Scanning Electron Microscopy

Samples of each MPC powder were attached to double-sided adhesive carbon tabs mounted on scanning electron microscope stubs, and then coated with chromium (K550X, Emitech, Ashford, UK). Scanning electron microscopy images were collected using a Zeiss Supra 40P field emission SEM (Carl Zeiss SMT Ltd., Cambridge, UK) at 2.00 kV. Representative micrographs were taken at 5000× magnification

### 2.7. Wettability of Milk Protein Concentrate Powders

Wettability was first measured using the method of GEA Niro [30] with a slight modification; 4 g of each sample was added to a beaker of water (25 °C) instead of 10 g. Wettability was also assessed using the method of Fitzpatrick [31] with some modifications; briefly, 10 g of powder was placed onto the surface of 250 mL of water (25 °C) in a 600 mL volume glass beaker. After 20 min, the remaining surface powder was carefully removed using a spatula. This powder was dried in an oven (102 °C) and its original water content was determined. Wettability (%; Equation (2)) was defined as:

(2) $$100\;\times \;\frac{mass\;of\;powder\;disappeared}{mass\;of\;initial\;powder}$$

### 2.8. Particle Size Distribution of Milk Protein Concentrate Dispersions

The particle size distribution of the MPC dispersions were measured using static light scattering (SLS) with a laser-light diffraction unit (Malvern Mastersizer 3000; Malvern Instruments Ltd, Worcestershire UK) equipped with a 300 RF lens. Particle and dispersant (i.e., water) refractive indices were set at 1.45 and 1.33, respectively. MPC powders were rehydrated (4% total solids, *w*/*w*) in ultrapure water under two different conditions: (a) high speed mixing for 30 s at 23 °C and (b) high speed mixing for 30 s at 50 °C. High speed mixing (3600 ± 100 rpm) was carried out using a solubility index meter (Labinco-BV, Breda, the Netherlands). Each sample was introduced into ultrapure water re-circulating at 20 °C in the dispersion unit (Hydro MV) at 1750 rpm. Size measurements were recorded as the median diameter (D_50_) and cumulative diameters (D_90_ and D_10_), whereby 50, 90, and 10% of the volume was smaller than the size indicated. Size distributions were obtained using polydisperse analysis. Measurements were recorded at a laser obscuration of 3–4% and all particle size measurements were performed in triplicate. 

### 2.9. Powder Solubility

MPC powders were dispersed in ultrapure water (23 °C; 4%, *w*/*w*, total solids) for 30 s using a solubility index meter (Labinco BV, Breda, the Netherlands). Aliquots (30 mL) of these solutions were then centrifuged at 3000× *g* for 10 min (23 °C) and the total solids content of the supernatant was then determined using a moisture analyser (CEM Smart System5™, 3100 Smith Farm Road, Matthews, NC, USA). The solubility of the powders was given by the total solids content of the supernatant expressed as a percentage of the total solids content of the initial dispersion.

### 2.10. Statistical Analysis

Measurements of the powder physical and rehydration characteristics were performed in triplicate. Analysis of variance (ANOVA; Tukey’s HSD) was carried out using the IBM SPSS (version 24, Armonk, NY, USA) statistical analysis package. The level of significance was determined at *p* < 0.05.

## 3. Results

### 3.1. Composition of Milk Protein Concentrate Powders

**Figure 1:**
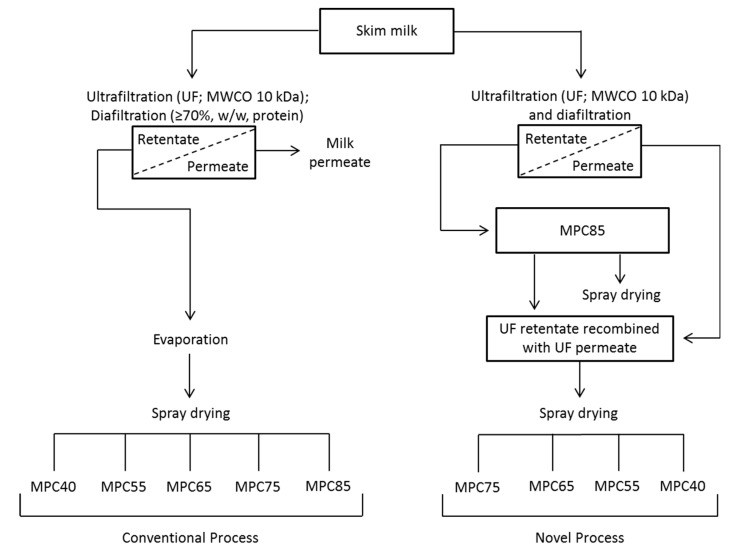
Process flow diagram of conventional and novel approaches for the production of milk protein concentrate (MPC) powders.

A process flow diagram comparing conventional milk protein concentrate (MPC) production with the novel approach used in this study is displayed in Figure 1, with the composition of the resultant MPC powders shown in Table 1. The recombination of the milk permeate with UF retentate resulted in a progressive decrease in the protein concentration of the MPC powders, with the powder moisture content tending to decrease with decreasing protein content. This was due to the higher viscosity of the feed prior to drying because of the higher protein content [32,33]. A high viscosity feed can result in larger spray droplets being produced during atomisation with reduced surface area available for the removal of moisture. Crowley [34] reported a moisture content of 4.6% (*w*/*w*) for MPC80 powder, compared to 3.4% (*w*/*w*) for MPC35. In the present study, significant (*p* < 0.05) differences in ash content were measured for the MPC powders, with the values ranging from 6.88% for MPC85 to 7.82% for MPC40 (Table 1). Deeth and Hartanto [35] reported similar ash results of 7.5 and 7.1% (*w*/*w*) for MPC42 and MPC85, respectively. In the present study, there was an increase in ash:protein with decreasing protein content, whereby the ash:protein ratio increased from 0.08 for MPC85 to 0.20 for MPC40 (Table 1). In a similar manner, Crowley [8] reported an ash:protein ratio of 0.23 for MPC35 compared to 0.10 for MPC85. 

### 3.2. Physical Properties of Milk Protein Concentrate Powders

#### 3.2.1. Powder Particle Size

**Figure 2:**
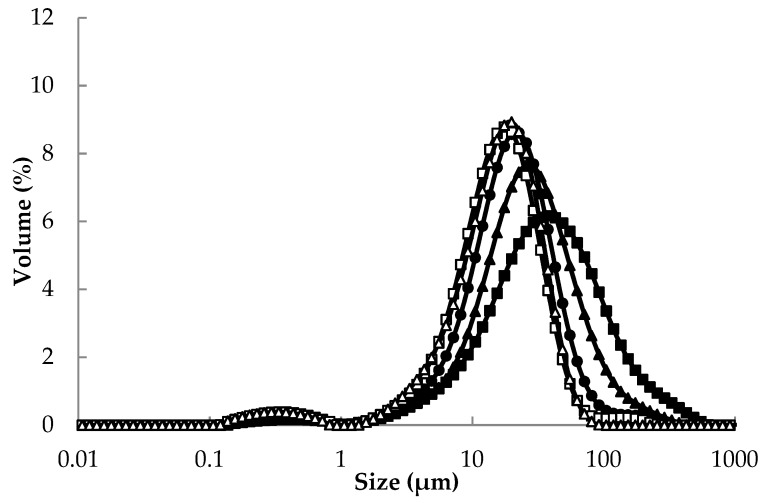
Particle size distribution of milk protein concentrate (MPC) 85 (■), MPC75 (▲), MPC65 (●), MPC55 (□), and MPC40 (∆) powders.

Powder particle size distribution analysis displayed a significant decrease in particle size with decreasing protein content (Figure 2); MPC85 had a D_[4,3]_ of 57.3 μm compared to 18.9 μm for MPC40 (Table 2). This is most likely caused by differences in the protein content of the concentrates prior to spray drying (as mentioned in Section 3.1), with high protein concentrates possessing a higher viscosity, thereby generating larger droplets during the atomisation step of spray drying [36]. Rupp [37] reported that the D_[4,3]_ of the MPC powder increased significantly from 31 to 50 μm with an increase in the protein content of the concentrate from 19 to 23% (*w*/*w*). Crowley [34] reported D_90_ values of 64.6 μm for MPC35 and 51.9 μm for MPC80 spray dried under similar conditions to the present study; however, this difference may be explained by the large differences in the concentrate total solids before spray drying (i.e., 35.5% *w*/*w* for MPC35 and 14.7% *w*/*w* for MPC85).

#### 3.2.2. Density

Particle, loose and tapped bulk density values for the MPC powders increased with decreasing protein content (Table 2). For instance, the particle density increased from 1.00 g/cm^3^ for MPC85 to 1.18 g/cm^3^ for MPC55, while tapped bulk density increased from 0.35 to 0.44 g/cm^3^, respectively. This finding is supported by the results of Crowley [34], who reported that particle density increased from 0.84 g/cm^3^ for MPC85 to 1.25 g/cm^3^ for MPC50, while tapped bulk density increased from 0.29 g/cm^3^ for MPC85 to 0.59 g/cm^3^ for MPC50. Eshpari [38] reported similar results to the present study with a particle density value of 1.07 g/cm^3^ for the MPC80 powder. There was a corresponding increase in both the interstitial and occluded air content of the powders as the density decreased. MPC85 powder had the lowest density (i.e., particle, loose, and tapped) and the highest interstitial (190 mL/100 g) and occluded (32.2 mL/100 g) air content, which may be accounted for by the greater powder particle size of this sample [39]. The increase in particle density with a decrease in the protein content could be accounted for by the concomitant increase in lactose in the powders. Furthermore, the MPC40 in the current study had a loose bulk density value of 0.40 g/cm^3^, which is lower than the value of 0.65 g/cm^3^ recorded by Fitzpatrick [28] for a commercial skim milk powder. This difference in bulk density may be due to the difference in the total solids content of the concentrate between the MPC40 sample (21.7%) and a typical commercial skim milk concentrate (e.g., 50%).

#### 3.2.3. Flowability

The flow index values obtained were similar for all powders (Table 3). For example, the flow index value for MPC65–85 was approximately 2.1. MPC40 had the highest flow index value of 2.6. However, as these values were all less than 4, the powders were categorised as cohesive according to the Jenike classification system for powder flowability. The poor flowability of the low-protein MPC sample (i.e., MPC40) is possibly related to the use of a two-fluid nozzle during spray drying, or the drying of this concentrate at a relatively lower total solids content than would be used for a typical commercial product with a similar protein content (e.g., skim milk). Crowley [34] reported that the flow index was reduced from 13.4 for MPC35 to 3.5 for MPC85, while Fitzpatrick [28] reported a flow index value of 6.1 for a commercial skim milk powder. The Hausner ratio (HR) values correlated with the flowability results, which demonstrated that high protein powders had poorer flowability than low protein powders. According to Turchiuli [40], a HR greater than 1.4 corresponds to a non-free flowing powder. Furthermore, the compressibility of MPC65-85 was significantly greater than that for both the MPC40 and MPC55 powders. This is most likely caused by the greater interstitial air content of the higher protein powders as these voids between powder particles would have been reduced considerably during compaction, resulting in a greater change in density.

#### 3.2.4. Microstructure

**Figure 3:**
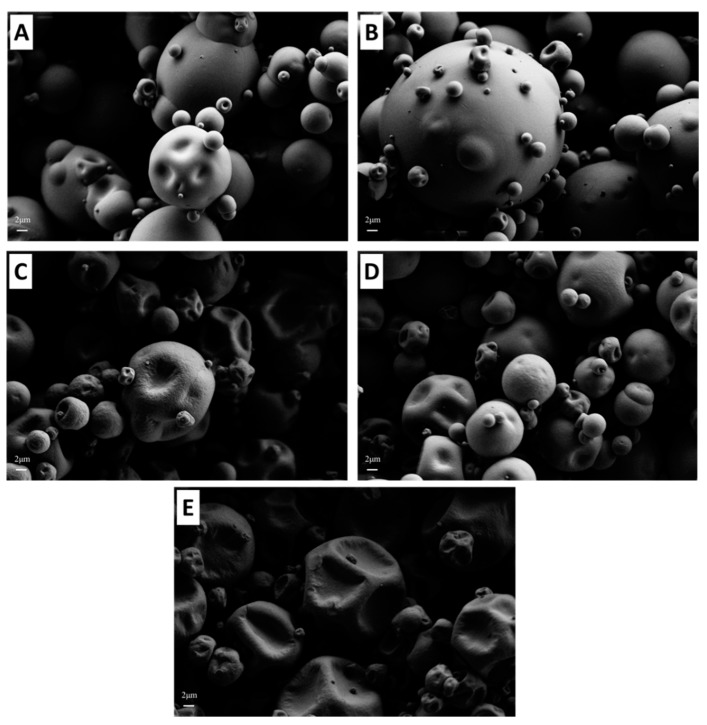
Scanning electron microscopy images of milk protein concentrate (MPC) 85 (**A**), MPC75 (**B**), MPC65 (**C**), MPC55 (**D**), and MPC40 (**E**) powders at 5000× magnification.

Scanning electron microscopy images of each MPC powder are shown in Figure 3. Low protein powders (e.g., MPC40) had a collapsed structure with wrinkled, concaved surfaces. However, for MPC75 and MPC85, the surface morphology changed significantly, with the surfaces of these powder particles appearing smoother and more dimpled. These results are supported by the findings of Kelly [41], who observed similar differences between the microstructures of spray-dried MPC powders (MPC35–90). The distinct differences in the microstructure of low and high protein MPC powders may be caused by several factors. Crowley [34] stated that lower protein MPC powders (i.e., MPC40) contained a lower volume of occluded air in comparison to higher protein MPC (i.e., MPC85), similar to the results of the current study, and likely accounts for the collapsed appearance of the particles. The smooth surface of high protein powders possibly arises from the compaction of casein micelles during the spray drying process [42]. Moreover, Sadek [43] and Tan [44] showed that protein type also plays an important role in powder particle morphology, with casein-dominant powder particles appearing more wrinkled compared to whey protein powders that possessed a spherical shape. Furthermore, spray drying temperatures can also affect particle morphology, with Tan [45] showing that an increase in drying inlet temperature could produce particles with wrinkled surfaces, while lower drying temperatures produced more spherical particles. 

### 3.3. Wettability of Milk Protein Concentrate Powders

Wettability analysis showed that MPC85 and MPC75 had the lowest wettability at 14.7% and 17.5% after 20 min, respectively, compared to approximately 47% for MPC40–65 (Table 3). Poor wetting behaviour of the MPC powders has previously been attributed to the hydrophobic, protein-rich surface of these ingredient powders [8,13]. Despite possessing similar protein content to skim milk powder, the MPC40 in the current study displayed poor wetting behaviour. Fitzpatrick [31] found that a skim milk powder completely wetted after 55 s at 20 °C, likely due to its large D_50_ value (132 µm) and a tapped bulk density of 0.55 g/cm^3^. MPC powders did not completely wet and sink within the time period measured; however, a visual difference was observed between samples (results not shown) with a smaller quantity of the low protein powders (i.e., MPC40 and MPC55) remaining on the surface of the water, with the water becoming more turbid, compared to the high protein powders (i.e., MPC75 and MPC85) that remained on the surface of the water and formed a surface film layer. This may also be accounted for by the differences in carbohydrate content between powders, with powders containing ≥22.8% lactose (*w*/*w*) likely being more hydrophilic, resulting in greater water transfer into and between proteins.

### 3.4. Dissolution and Solubility of Milk Protein Concentrate Powders

**Figure 4:**
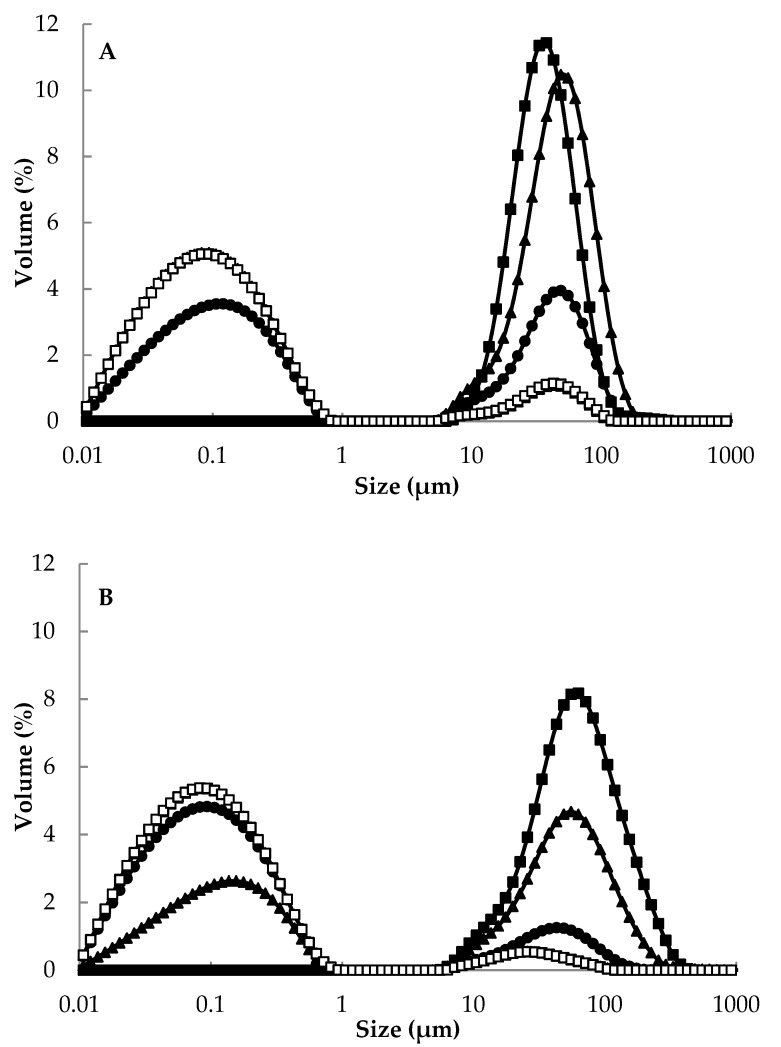
Particle size distribution of milk protein concentrate (MPC) 85 (■), MPC75 (▲), MPC65 (●), MPC55 (□), and MPC40 (∆) powders after reconstitution in ultrapure water at (**A**) 23 °C and (**B**) 50 °C.

The particle size distribution data indicated the presence of large, poorly dispersible particles in high protein MPC powders (Figure 4). This was most apparent for MPC85 and MPC75 when dispersed in water at 23 °C as they exhibited monomodal size distribution in the range 5–100 μm (Figure 4A). Dispersion of powder particles is considered the rate limiting stage in the rehydration of MPC [7], and this is most likely caused by protein–protein (e.g., hydrophobic) interactions between casein micelles in close proximity and the low concentration of lactose facilitating close packing [17,46]. On the other hand, bimodal distributions were observed for MPC40–65, which suggests the presence of both casein micelles (<1 µm) and primary powder particles (>1 µm).

The volume of primary particles generally decreased with the reducing protein content of the powders. MPC55 and MPC40 displayed the highest dispersibility, which corresponded to a small volume of large particles in the range of 5–100 μm, and a larger volume of sub-micron (<1 μm) particles. Additionally, the D_[4,3]_ value generally decreased as the protein content of the powders was reduced, e.g., 51.7 μm for MPC75 compared with 4.25 μm for MPC40 when the samples were reconstituted at 23 °C (Table 4). The target particle size profile for a rehydrated MPC would be a monomodal distribution in the size range of casein micelles, (i.e., <1 μm). It has been reported that a mean particle size of 0.08–0.2 μm represents the presence of casein micelles, providing evidence that the hydration of powder particles has taken place [10,47].

Reconstitution of MPC85 and MPC75 powder in water at 50 °C reduced the volume of primary powder particles, but resulted in the occurrence of some particles with a size >100 μm (Figure 4B). This may be accounted for by powder particle swelling caused by greater water uptake and hydration at 50 °C than at 23 °C; however, even though hydration occurred, it is suggested that complete particle dissociation did not occur as a large volume of particles remained in the 10–500 μm size range. The swelling stage of powder rehydration had previously been observed by Gaiani [12] during the rehydration of micellar casein powder, whereby swelling was recorded as a peak in particle size following powder wetting. The short period of reconstitution (30 s) in 50 °C water appears to have been sufficient to allow wetting of high protein powders to occur, but insufficient to enable complete dispersion of powder particles. Conversely, MPC40–65 powders had lower D_[4,3]_ values when dispersed at 50 °C, compared to at 23 °C, indicating that after water sorption, the powder particles began to dissociate. The solubility was greater for the low protein powders, (i.e., MPC40 and MPC55) in comparison to the higher protein powder (i.e., MPC85; Table 3). The MPC40–65 powders all displayed solubility of approximately 98%, compared with just 83% for MPC85. These results support those recorded during the particle size distribution analysis; high protein MPC powders (75–85%, *w*/*w*) displayed poor dispersion and solubility properties in water. (Note: Lactose crystallisation, which is an important factor to consider in relation to the solubility of the MPC powders, did not occur in the current study (results not shown). Maidannyk [24] reported that MPC powders, ranging in protein content from 40–80% (*w*/*w*), did not show lactose crystallisation in their amorphous state following spray drying, but this process did occur for MPC40, 50, and 60 powders stored at high relative humidity).

## 4. Conclusions

This study provided new information on the physical properties of milk protein concentrate powders prepared through the novel combination of milk permeate and high protein UF retentate to create MPC powders at different protein contents, but with comparable physical and rehydration characteristics to those produced by conventional direct UF concentration and drying. Powder particle size decreased with a decrease in the protein content of the concentrate, most likely due to differences in concentrate viscosity. Decreasing the protein content also brought about an increase in bulk, tapped, and particle density of the MPC powders. The wetting and dispersion of the powders were improved by decreasing the protein and increasing the lactose content of the blends. The rehydration and physical properties of the MPC powders were significantly altered by changes in concentrate composition, but did not appear to be affected by the method of manufacture (i.e., concentrate standardisation with milk permeate compared with direct membrane concentration).

## Figures and Tables

**Table 1:** Composition of milk protein concentrate (MPC) powders.

MPC	Protein	Lactose	Fat	Ash	Moisture	Ash:Protein
(%, *w*/*w*)						
MPC85	84.7 ± 0.9	1.37	2.07	6.88 ^a^ ± 0.1	6.68 ^a^ ± 0.3	0.08
MPC75	74.1 ± 0.8	12.6	1.59	6.99 ^b^ ± 0.0	5.19 ^b^ ± 0.1	0.09
MPC65	63.6 ± 0.7	22.8	1.34	7.17 ^c^ ± 0.0	5.49 ^b^ ± 0.1	0.11
MPC55	53.7 ± 1.3	33.4	1.17	7.43 ^d^ ± 0.0	5.09 ^b^ ± 0.0	0.14
MPC40	38.9 ± 0.6	48.2	0.87	7.82 ^e^ ± 0.0	4.59 ^c^ ± 0.0	0.20

^a–e^ Values within a column not sharing common superscripts differ significantly (*p* < 0.05).

**Table 2:** Particle density (p_p_), loose bulk density (p_b_), tapped bulk density (p_t_), volume of interstitial air (V_ia_), volume of occluded air (V_oa_), particle size below which 90% of material volume exists (D_90_), and the volume weighted mean particle diameter (D_[4,3]_) values for milk protein concentrate (MPC) powders.

MPC	p_p_	p_b_	p_t_	V_ia_	V_oa_	D_90_	D_[4,3]_
	(g/cm^3^)			mL/100 g		μm	
MPC85	1.00 ^a^ ± 0.0	0.29 ^a^ ± 0.0	0.35 ^a^ ± 0.0	190 ^a^ ± 7.8	32.2 ^a^ ± 0.1	127 ^a^ ± 4.5	57.3 ^a^ ± 2.9
MPC75	1.08 ^b^ ± 0.0	0.32 ^b^ ± 0.0	0.38 ^b^ ± 0.0	173 ^a^ ± 5.6	25.5 ^b^ ± 0.4	76.1 ^b^ ± 1.4	37.5 ^b^ ± 0.7
MPC65	1.14 ^c^ ± 0.0	0.34 ^c^ ± 0.0	0.41 ^c^ ± 0.0	155 ^b^ ± 3.1	20.5 ^c^ ± 0.8	47.4 ^c^ ± 1.0	25.5 ^c^ ± 0.4
MPC55	1.18 ^d^ ± 0.0	0.39 ^d^ ± 0.0	0.44 ^d^ ± 0.0	141 ^b^ ± 10	17.5 ^d^ ± 1.1	36.3 ^d^ ± 0.8	19.9 ^d^ ± 0.6
MPC40	1.14 ^c^ ± 0.0	0.40 ^d^ ± 0.0	0.43 ^cd^ ± 0.0	143 ^b^ ± 0.8	21.1 ^c^ ± 0.7	35.9 ^d^ ± 0.3	18.8 ^d^ ± 0.2

^a–d^ Values within a column not sharing common superscripts differ significantly (*p* < 0.05).

**Table 3:** Flow and rehydration (wettability and solubility) properties of milk protein concentrate (MPC) powders.

MPC	i	JC	CI (%)	HR	Wettability (%)	Solubility (%)
MPC85	2.1 ± 0.1	Cohesive	41.2 ^a^ ± 1.5	1.71	14.7 ^a^ ± 1.8	83.0 ^a^ ± 2.2
MPC75	2.1 ± 0.0	Cohesive	42.1 ^a^ ± 0.7	1.73	17.5 ^a^ ± 2.0	92.9 ^b^ ± 1.6
MPC65	2.0 ± 0.3	Cohesive	41.9 ^a^ ± 2.6	1.73	49.3 ^b^ ± 1.1	98.0 ^c^ ± 1.3
MPC55	2.2 ± 0.2	Cohesive	35.0 ^b^ ± 1.3	1.55	48.3 ^b^ ± 1.1	98.5 ^c^ ± 1.1
MPC40	2.6 ± 0.2	Cohesive	32.4 ^b^ ± 1.8	1.50	48.3 ^b^ ± 0.9	98.1 ^c^ ± 0.8

^a–d^ Values within a column not sharing common superscripts differ significantly (*p* < 0.05). i = flow index, JC = Jenike classification, CI = compressibility index, HR = Hausner ratio.

**Table 4:** Mean particle size of milk protein concentrate (MPC) dispersions after high speed mixing at 23 °C and 50 °C.

**MPC**	D_90_ (μm)		D_[4,3]_ (μm)	
	23 °C	50 °C	23 °C	50 °C
MPC85	68.9 ^a^ ± 5.4	156 ^a^ ± 11	40.7 ^a^ ± 2.9	76.4 ^a^ ± 4.3
MPC75	92.6 ^b^ ± 4.2	98.2 ^b^ ± 2.2	51.7 ^b^ ± 1.9	36.7 ^a^ ± 3.5
MPC65	59.7 ^c^ ± 2.1	25.6 ^c^ ± 11	18.3 ^c^ ± 1.6	6.68 ^a^ ± 1.9
MPC55	13.1 ^d^ ± 4.6	0.39 ^d^ ± 0.0	4.57 ^d^ ± 0.3	1.98 ^b^ ± 0.2
MPC40	6.30 ^e^ ± 5.8	0.41 ^d^ ± 0.1	4.25 ^d^ ± 0.3	2.06 ^b^ ± 0.4

^a–d^ Values within a column not sharing common superscripts differ significantly (*p* < 0.05). D_90_ = the size of particles below which 90% of the sample lies. D_[4,3]_ = volume weighted mean diameter.
